# Heavy metal bioaccumulation and antioxidative responses in *Cardaminopsis arenosa* and *Plantago lanceolata* leaves from metalliferous and non-metalliferous sites: a field study

**DOI:** 10.1007/s10646-013-1129-y

**Published:** 2013-10-02

**Authors:** Aleksandra Nadgórska-Socha, Bartłomiej Ptasiński, Andrzej Kita

**Affiliations:** 1 Department of Ecology, University of Silesia, Bankowa 9, 40-007 Katowice, Poland; 2 Analytical Spectroscopy Research Group, Institute of Chemistry, University of Silesia, Szkolna 7, 40-007 Katowice, Poland

**Keywords:** Heavy metals, Antioxidants, Antioxidant enzymes, *Cardaminopsis arenosa*, *Plantago lanceolata*

## Abstract

The purpose of this study was to determine the concentrations of heavy metals (cadmium, lead, zinc, copper, iron and manganese) in soil, their bioavailability and bioaccumulation in plants leaves. This study also examined their influences on the antioxidant response of the plants *Cardaminopsis arenosa* and *Plantago lanceolata* grown in metal-contaminated and non-contaminated soils. The activities of guaiacol peroxidase and superoxide dismutase and the levels of antioxidants such as glutathione, proline and non-protein thiols were measured. Concentrations of the examined metals were several to thousands of times lower in the potentially bioavailable fraction than in the acid-extracted fraction of the soil. Similar mode of antioxidant responses in plant leaves of metalliferous populations indicates the tolerance of plants towards heavy metals. However POD and GSHt had a particularly strong role in defense reactions, as their increase was the most common reaction to heavy metal contamination.The levels of Zn, Cd and Pb in the leaves of *C. arenosa* better reflected metal concentrations in the metalliferous and non-metalliferous soil than the determined metal concentrations in *P. lanceolata*. Bioaccumulated Zn, Cd and Pb concentrations were above or in the ranges mentioned as toxic for plant tissues and therefore the studied plants have potential for use in phytostabilization.

## Introduction

Heavy metal contamination is a worldwide problem that results in bioaccumulation in the food chains, posing a direct threat to wildlife and human health. Although naturally present in soils, they rarely occur at toxic levels. Heavy metal contamination therefore largely is anthropogenic—a result of industrial manufacturing, agriculture, combustion of fossil fuels, and road traffic, among other causes (Przedpełska and Wierzbicka 2007; Massa et al. 2010; Serbula et al. 2012). This can be exceptionally high in the vicinity of smelting operations and near mine tailings, which are materials left over after the process of separating the valuable fraction from the uneconomic fraction of an ore (Probst et al. 2009; Bothe 2011).

Environmental quality monitoring using biological material commonly is accepted as a reliable and affordable way to obtain information on heavy metal contamination. The main advantage is the opportunity for long-term comparison without the need for expensive equipment. Such biomonitoring studies include research on metal bioaccumulation in vegetation found in contaminated areas, and also for the purpose of finding plants which may be used for phytoremediation (Massa et al. 2010).

Contaminated sites can be considered as reservoirs of native plants with the ability to tolerate or bioaccumulate heavy metals to different ranges. Indigenous plants, very often ruderals, which first colonize disturbed lands, can be valuable bioindicators and bioaccumulators of heavy metals in contaminated areas (Kovács 1992; Massa et al. 2010). Taxa colonizing soils rich in heavy metals are either obligate or facultative metallophytes which have evolved mechanisms to tolerate high concentrations of heavy metals. As such, they are very good biological specimens for research on adaptation to harsh edaphic conditions (Przedpełska and Wierzbicka 2007; Słomka et al. 2008).

We already know several efficient mechanisms for restricting excessive metal concentration in metabolic compartments of plant cells. However, as some metal ions remain in the cytoplasm and induce oxidative stress via the generation of reactive oxygen species (ROS), it is the effectiveness of plants antioxidant defenses that is most crucial for their resistance to metals. Plants have developed scavenging systems that control ROS using non-enzymatic antioxidants, such as glutathione, ascorbate and carotenoids, as well as an enzymatic anti-oxidative system. Activities of anti-oxidative enzymes such as superoxidase dismutase (SOD), glutathione peroxidase (GPX), catalase (CAT) and guaiacol peroxidase (POD), often have been examined in research on heavy metal antioxidant defenses (Pongrac et al. 2009; Kafel et al. 2010; Boojar and Tavakkoli 2011).

This paper examines *Plantago lanceolata* and *Cardaminopsis arenosa* (synonym: *Arabidopsis* *arenosa*) samples taken from contaminated and non-contaminated sites. *P. lanceolata*, a member of the Family *Plantaginaceae*, is a common roadside plant that is widely distributed throughout the world. *C*. *arenosa* is a member of the Family *Brassicaceae* that is very often found in disturbed habitats such as forest edges, road sides, railroad tracks, river banks as well as in grassy and sandy areas. These taxa can also be found among metallophytes occurring in areas contaminated with heavy metals (Przedpełska and Wierzbicka 2007; Gostin 2009; Szarek-Łukaszewska and Grodzińska 2011). Some researchers have highlighted the advantages of studies on such plants growing in natural conditions. The advantage is the direct documentation of microevolutionary processes, i.e., genotype-level adaptation and its plasticity and ecological status (Ernst 2006; Słomka et al. 2008).

In the literature, we find only few reports about *C. arenosa* or *P. lanceolata* tolerance to heavy metals (Szarek- Łukaszewska and Niklińska 2002; Dimitrova and Yurukova 2005; Przedpełska and Wierzbicka 2007; Kurteva 2009; Orłowska et al. 2012). *C. arenosa* and *P. lanceolata* were chosen because of their frequent occurrence on metalliferous soils and more frequent occurrence than other metallophytes from study areas on non-metalliferous soil. Among the representatives of *Brassicaceae* and *Plantaginaceae*, they are species related to hyperaccumulators, *Arabidopsis halleri* (Zn, Pb, Cd hyperaccumulator), *Plantago almogravensis* (Al hyperaccumulator), and *Plantago orbignyana* (Pb accumulator). This is why *C. arenosa* and *P. lanceolata* may be interesting for studying heavy metal bioaccumulation and ecophysiological response (Przedpełska and Wierzbicka 2007; Braquinho et al. 2007; Bech et al. 2012).

The objective of this study was the comparison of heavy metal [Cd, Zn, Pb, Mn, Fe, Cu] bioaccumulation patterns in leaves of *C. arenosa* and *P. lanceolata* in order to evaluate environmental quality monitoring and environmental risk assessment. The aim of this study was also to examine the activity of selected antioxidant enzymes [superoxidase dismutase (SOD, EC 1.15.1.1), guaiacol peroxidase (POD, EC 1.11.1.7)] and the levels of antioxidants [glutathione, proline and non-protein thiols] in the leaves of *C*. *arenosa* and *P. lanceolata*, if investigated physiological parameters in *C. arenosa* and *P. lanceolata* plants from the contaminated sites vary from the ones collected at non-contaminated sites. Knowledge about the tolerance of plants to heavy metal toxicity is required before considering their possible application in soil phytostabilization and revegetation of mining areas contaminated with heavy metals. We also studied the potential bioavailability of metals in the soil from contaminated areas and a non-contaminated control area. The following hypothesis was evaluated: heavy metal contamination contributes to changes in the antioxidant responses in both species within the contaminated sites, and in comparison to plants from the non-contaminated area.

## Materials and methods

### Study areas

The study areas are located in the southern part of Poland. We chose four areas contaminated with heavy metals (site code M- metalliferous): (1) vicinity of the zinc plant “Miasteczko Śląskie” (M1), (2) vicinity of a former metal smelting operation “Szopienice” in Katowice (M2), (3) a zinc-lead (calamine) site in Dąbrowa Górnicza (M3), and (4) a calamine waste heap in Bolesław near Olkusz (M4). M3 and M4—calamine areas, were connected with ore mining and processing of zinc ores called calamines. Soil and plant material were also collected from grassland and carpet communities (consisted of species, which tolerate mechanical damages e.g., *Lolio*-*Plantaginetum*) on the perimeter of the Pazurek Nature Reserve near Olkusz, selected as a contamination-free control area (Site code “NM”-non-metalliferous). We selected NM site thanks to data from a previous study conducted in the Pazurek Nature Reserve (Nadgórska-Socha unpubl., Kandziora-Ciupa et al. 2013). The areas studied near smelting operations were situated in the heavily industrialized region of Upper Silesia. The first calamine site (M3) was situated in Silesia and the calamine heap (M4) and control area were located in Małopolska Province. Geographic locations and ecological backgrounds of the investigated natural populations of *C. arenosa* and *P. lanceolata* are presented in Table 1. The Pazurek Nature Reserve perimeter, selected as a control area, is located nearby the contaminated sites (66 km from M1; 46 km from M2, 25 km from M3 and 15 km from M4) and was selected because of similar climatic conditions to the other study areas. Soil pH was lower than in metalliferous sites because of the pine forest in the vicinity. It was also used as control due to low zinc and cadmium concentrations and Pb concentration much lower than in the contaminated sites. Also, N and organic matter content were similar, especially to M3 and M4 sites (Table 1).

**Table 1:** Geographic location and ecological background of the *C. arenosa* and *P. lanceolata* investigated natural populations

Name	Habitat	Origin of contamination	Latitude	Longitude	Vegetation (dominant species)
Miasteczko Śląskie (M1)	Roadside	Metallurgic acivity since 1967	50°31′22.655″N	18°56′8.699″E	*Agrostis capillaris* *Calamagrostis epigejos* *Silene vulgaris* *Cardaminopsis arenosa* *Melandrium album* *Plantago lanceolata*
Katowice-Szopienice (M2)	Grassland	Metallurgic acivity since 1834	50°15′29.65″N	19°6′42.88″E	*Agrostis capillaris* *Festuca rubra* *Silene vulgaris* *Cardaminopsis arenosa*
Dąbrowa Górnicza (M3)	Forest edge and grassland	Mining activities during 19th century	50°18′58.859″N	19°18′28.62″E	*Betula pendula* *Festuca ovina* *Silene vulgaris* *Plantago lanceolata*
Bolesław (M4)	Grassland	Mining activities since 19th century	50°17′11.472″N	19°28′5.231″E	*Silene vulgaris* *Cardaminopsis arenosa* *Festuca ovina*
Pazurek (NM)	Forest edge	–	50°19′58.74″N	19°35′59.82″E	*Pinus sylvestris* *Plantago lanceolata*

### Soil and plant material

We sampled the plant material and soil in early June 2009. The soil samples were taken from topsoil (0–15 cm in depth), as heavy metal contamination mainly was concentrated in the topsoil and the roots of the selected plants do not grow any deeper. Each sampling site covered 100 m^2^ within which leaf and soil samples were randomly collected. Ten (10) soil sub-samples and 20 individual plants of each species—(*C. arenosa* and *P. lanceolata*) were taken from each sampling area and pooled into one sample of each species per site. The plants were taken during the flowering stage, the highest metabolic point during the plant life cycle (Dazy et al. 2008). After collection plants samples were placed in plastic bags, deposited in ice, immediately transported to the laboratory and then frozen at −80 °C until analysis. The heavy metal content determinations and biochemical analyses were conducted on the leaves of the plants. Each analysis was performed in five replicates.

### Analysis of metal concentration in soil and plant samples

The concentrations of cadmium, lead, zinc, copper, iron and manganese were analyzed. The metal content of the soil was estimated according to the method by Bouwman et al. (2001) and Ostrowska et al. (1991), and previously described in details (Nadgórska-Socha et al. 2013). Metals were extracted from air-dried samples of soil using 0.01 M CaCl_2_ (potentially bioavailable elements) or using 2 M HNO_3_ (extracted elements). Soil pH was measured using a 1:2.5 soil to water ratio. Organic matter content (expressed in  %) was measured following the methods of Ostrowska et al. (1991). The levels of C, N, and S were measured with a CNS analyzer (Variomax CNS, Elementar Analysensysteme GmbH, Germany).

In order to determine heavy metal concentrations, plant material was washed in tap and distilled water and dried at 105 °C. After wet mineralization [previously described in detail (Nadgórska-Socha et al. 2013)], metal contents (Cd, Pb, Zn, Cu, Fe and Mn) were measured by inductively coupled plasma emission spectroscopy (Spektroflame-M spectrophotometer, ICP Spectro Analytical Instruments, Germany). The quality of the analytical procedure was checked using a reference material (Certified Reference Material CTA-OTL-1 Oriental Tobacco Leaves, Department of Analytical Chemistry, Institute of Nuclear Chemistry and Technology, Poland) with the same quantities as samples.

### Analysis of the biochemical parameters of the plants

Crushed plant parts were homogenized in a 100 mM phosphate buffer (pH 6.8) for the analysis of POD activity (1:7 ratio) and centrifuged at 12,000×*g* for 20 min. The entire procedure was carried out at 4 °C. The activity of POD was measured at 470 nm according to Fang and Kao (2000) using guaiacol as the substrate, and it was expressed in μmol of tetra-guaiacol per min^−1^per mg of protein^−1^.

The analysis of superoxide dismutase (SOD) was performed in a buffer with 3 mM MgSO_4_, 1 mM dithiotreitol (DTT), 3 mm EDTA (1:5 ratio) and centrifuged at 12,000×*g* for 20 min. The entire procedure was carried out at 4 °C. The reaction was measured spectrophotometrically at 560 nm according to Beauchamp and Fridovich (1971). One unit of SOD was defined as the amount of enzyme activity that was able to inhibit by 50 % the photoreduction of nitroblue tetrazolium (NBT) to blue formazan (Beauchamp and Fridovich 1971). This method was chosen because the method was supposed to be suitable and it is still being used (sometimes with modifications) in recent ecophysiological studies.

To measure the contents of non-protein thiols, plant material was homogenized in a 5 vol/g mixture containing 5-sulphosalicylic acid (2 g per 100 mL), and 1 mM EDTA and sodium ascorbate (0.15 g per 100 mL). The number of non-protein SH groups was established based on a curve prepared using l-cysteine and expressed as nmol—SH g^−1^ fresh weight (Mass et al. 1987).

The acid-ninhydrin method was used to determine proline content (Bates et al. 1973). Plant material was homogenized in sulfosalicylic acid (3 g per 100 mL). The proline content expressed in μmol proline g^−1^ fresh weight was calculated as described by Bates et al. (1973).

The content of total glutathione (GSHt) was measured following the methods of Anderson (1985), and protein content was measured following the methods of Bradford (1976), using the appropriate standard curves of oxidized glutathione and bovine standard albumin solutions, respectively.

To detect the glutathione concentration, plant leaves were homogenized in TCA (trichloroacetic acid, 5 g per 100 mL) and 0.125 mM phosphate buffer (pH 6.3) with 6.3 mM EDTA, and then were centrifuged at 10,000×*g* for 10 min at 4 °C. The linear changes in the absorbance of the reaction mixtures were measured at 412 nm and GSHt was expressed as μmol GSH g^−1^ fresh weight.

### Statistical assessment

The biochemical parameter data and metal content were checked for normality and equality of variance, and when necessary the data was log transformed. One-way ANOVA was carried out to compare the difference of means from various sampling sites and significant statistical differences were established using Tukey’s test, *p* < 0.05 [ANOVA; Statistica version 10 package, StatSoft, Inc. (2011)]. We also calculated the linear correlation coefficient between the metal concentrations in separate soil extractants and in the leaves of plants, as well as between the metal concentrations and biochemical parameters in the leaves of *C. arenosa* and *P. lanceolata.*

CANOCO 4.5 was used to carry out Principal Component Analysis (PCA) (Ter Braak and Šmilauer 2002). Principal Component Analysis assessed the similarities and relations between biochemical parameters and elemental content in the plants.

## Results

### The bioavailability of metals in soil

There was a clear difference between the concentration of metals in the fraction of soil extracted with HNO_3_ and the fraction of soil extracted with CaCl_2_ (Table 2). The highest concentration of Zn was found in the acid extracted fraction of soil. The highest acid extracted concentrations of Cd (301.2 mg kg^−1^), Zn (70,445.8 mg kg^−1^) and Cu (74.1 mg kg^−1^), Fe (2,632.5) were in the soil collected in M3 site, while Pb (4,230.9 mg kg^−1^) concentration was the highest in M4 site.

**Table 2:** The concentrations of selected metals in fractions of the soils extracted with HNO_3_ and CaCl_2_ [mg kg^−1^], organic matter content [%], N, C, S [%] and pH value from investigated sites

Metal/stand	M1	M2	M3	M4	NM
Cd (HNO_3_)	6.1 a	90.8 b	301.2 d	175.6 c	2.7 e
Zn (HNO_3_)	2,878.3 a	8,403.3 b	70,445.8 c	68,570.8 c	358.8 d
Pb (HNO_3_)	959.1 a	394.7 b	3,619.1 d	4,230.9 c	123.1 e
Cu (HNO_3_)	11.2 a	34.3 b	74.1 d	8.5 c	12.9 a
Mn (HNO_3_)	65.7 a	236.7 b	768.0 c	779.2 c	64.7 a
Fe (HNO_3_)	1,273.9 a	1,740.8 b	2,632.5 d	2,027.0 c	1,380.3 e
Cd (CaCl_2_)	0.2 a	11.2 b	2.3 d	1.2 c	0.6 a
Mn (CaCl_2_)	5.1 a	2.3 b	3.1 d	2.1 c	1.7 e
Zn (CaCl_2_)	32.4 a	374.0 b	22.0 c	34.8 a	11.5 d
Organic matter content	2.1 a	3.1 a	12.0 b	14.0 b	11.9 b
pH	7.5 a	7.3 b	7.4 a	7.3 b	5.6 c
N	0.1 a	0.1 a	0.3 b	0.4 c	0.3 b
C	1.1 a	1.5 b	8.8 c	11.3 d	4.4 e
S	0.03 a	0.07 b	0.02 c	0.03 a	0.04 d

The *different letters* denote significant differences between the particular metal concentrations in the fraction extracted with HNO_3_, extracted with CaCl_2_, organic matter contents and pH values (*p* < 0.05)

Metal concentrations were from 8 times (Cd in M2) to about 3,200 times (Zn in M3) lower in the bioavailable fraction (CaCl_2_ extraction) in metalliferous sites. Potentially available Cd ranged from 0.76 to 21 % of Cd extracted with HNO_3_ (Table 2), and thus proved to be the most bioavailable element. The bioavailable Mn ranged from 0.3 to 7.7 % and bioavailable Zn from 0.03 to 4.5 % of the total amount extracted with HNO_3_.

The concentrations of other metals were under the detectable limit of the apparatus. The examined soil from all areas had rather low organic matter and C content, especially in the vicinity of contamination emitters. N content in soil of M3 and M4 sites was similar to NM area (Table 2). The pH values were above 7 at the contaminated sites and pH was in the acidic range only in the control area (NM) (Table 2).

### Heavy metal concentration in plants

The mean values of heavy metal concentrations in leaves of investigated plants were found in the following descending order—Zn > Fe > Pb > Mn ≥ Cd > Cu. *C. arenosa* bioaccumulated higher amounts of Zn, Cd, Pb, Mn in the leaves than *P. lanceolata.* Bioaccumulated amounts of Fe (except M3) and Cu were similar in the leaves of these two species (Tables 3, 4).

**Table 3:** The concentrations of heavy metals (mg kg^−1^ d.w.) in the leaves of *C. arenosa*

Stand metal	M1	M2	M3	M4	NM
Cd	45.6 a	41.5 a	12.9 c	100.2 b	1.4 d
Pb	256.6 a	30.8 b	94.5 d	70.8 c	6.2 e
Zn	3,054.1 a	5,438.7 b	2,828.0 ac	2,594.9 c	256.9 d
Cu	4.8 a	2.3 b	3.9 d	0.8 c	2.2 b
Fe	274.1 a	365.7 b	2,588.3 d	170.5 c	279.8 a
Mn	62.3 a	12.9 b	74.3 c	11.8 b	23.8 d

The *different letters* denote significant differences between the metal concentration in plants from metalliferous and non-metalliferous populations (*p* < 0.05)

**Table 4:** The concentrations of heavy metals (mg kg^−1^ d.w.) in the leaves of *P. lanceolata*

Metal/stand	M1	M2	M3	M4	NM
Cd	13.8 a	9.1 b	5.7 b	7.1 b	1.0 c
Pb	107.2 a	121.3 b	67.5 d	19.5 c	0.03 e
Zn	372.9 a	209.8 b	420.1 a	219.1 b	101.3 c
Cu	6.2 a	3.5 a	1.2 b	4.1 a	3.2 c
Fe	232.7 a	208.2 a	1,065.6 b	175.7 a	218.7 a
Mn	13.5 a	7.0 b	22.7 c	9.5 a	6.1 b

The *different letters* denote significant differences between the metal concentration in plants from metalliferous and non-metalliferous populations (*p* < 0.05)

The highest Zn concentration (5,438.6 mg kg^−1^) was found in *C. arenosa* leaves collected in M2 site, the highest Pb content (256.55 mg kg^−1^) in M1 site and the highest Cd content (100.15 mg kg^−1^) in M4. Fe (2,588.3 mg kg^−1^) and Mn (74.3 mg kg^−1^) content were the highest in the M3 area (Tables 3, 4)

We noted a strong positive correlation between concentrations of Zn (correlation coefficient = 0.81) and Mn (correlation coefficient = 0.92) examined under CaCl_2_ extraction and in the leaves of *C. arenosa*. We also found a positive correlation between concentrations of Mn (correlation coefficient = 0.48) and Fe (correlation coefficient = 0.72) under HNO_3_ extraction and in the leaves of *P. lanceolata*. The range of other correlation coefficients was statistically insignificant.

### The biochemical status of the plants

An increase in GSHt level was found in leaves of *C. arenosa* and *P. lanceolata* collected from the most contaminated areas in comparison to its content in plants from the (NM) area. Only in the leaves of *P. lanceolata* from M1 site and in the leaves of *C. arenosa* from M3 was the GSHt content comparable to the level in the leaves of this species from the NM area.

**Fig. 1:**
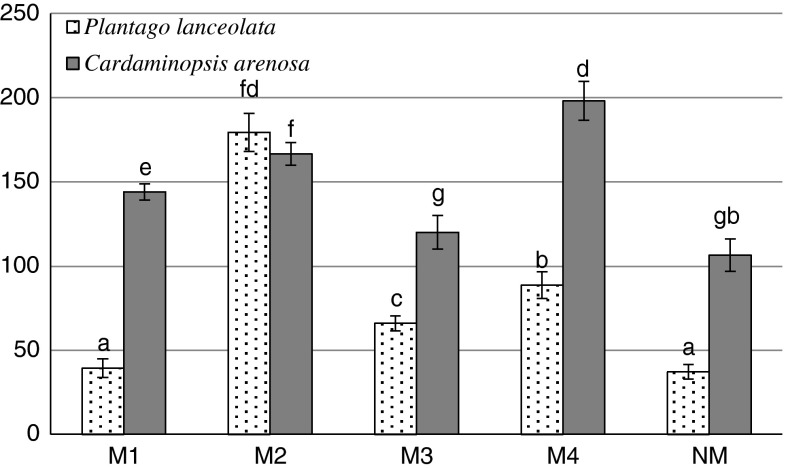
GSHt content in *P. lanceolata* and *C. arenosa* leaves

The highest GSHt concentration (198 μmol GSH g^−1^ fresh weight) was recorded in the leaves of *C. arenosa* (M4), about two times more than in the plants from the NM area (Fig. 1). The total glutathione pool positively correlated with Zn and Cd content in *C. arenosa* leaves and with Pb contentin *P. lanceolata* leaves. In addition, a negative correlation was found between Fe, Mn, Cu content and glutathione concentration in *C. arenosa* leaves (Tables 5, 6).

**Table 5:** The correlation coefficients between metal concentration and antioxidant measurements in the leaves of *Cardaminopsis arenosa* plants (*p* < 0.05)

	PRO	GSHt	SH	SOD	POD
Cd	0.52	0.92	−0.58	NS	−0.42
Zn	NS	0.51	NS	NS	0.40
Pb	0.43	NS	NS	NS	NS
Cu	−0.10	−0.51	NS	0.50	0.68
Fe	−0.79	−0.42	−0.46	0.95	0.76
Mn	−0.27	−0.52	NS	0.85	0.74

*NS* not significant

**Table 6:** The correlation coefficients between metal concentration and antioxidant measurements in the leaves of *Plantago lanceolata* plants (*p* < 0.05)

	PRO	GSHt	SH	SOD	POD
Cd	−0.59	NS	NS	NS	0.73
Zn	−0.74	NS	NS	NS	0.41
Pb	−0.45	0.53	0.51	NS	0.86
Cu	NS	NS	0.61	−0.67	0.59
Mn	−0.53	NS	NS	0.76	NS
Fe	NS	NS	NS	0.80	−0.46

*NS* not significant

**Fig. 2:**
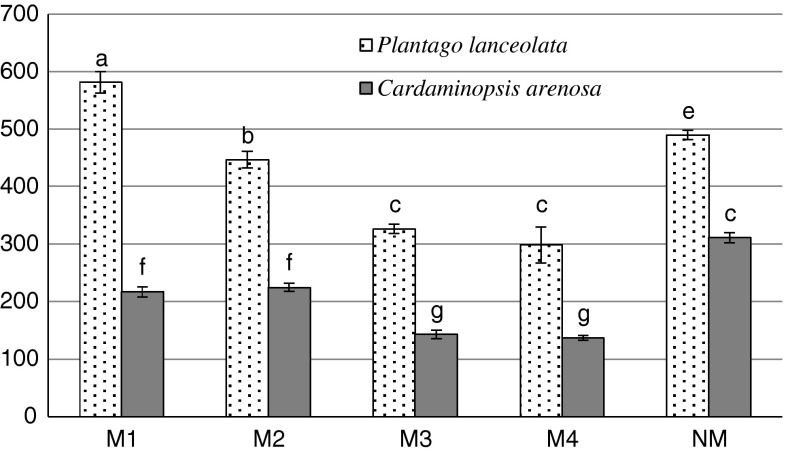
Non-protein -SH group content in *P. lanceolata* and *C. arenosa* leaves

**Fig. 3:**
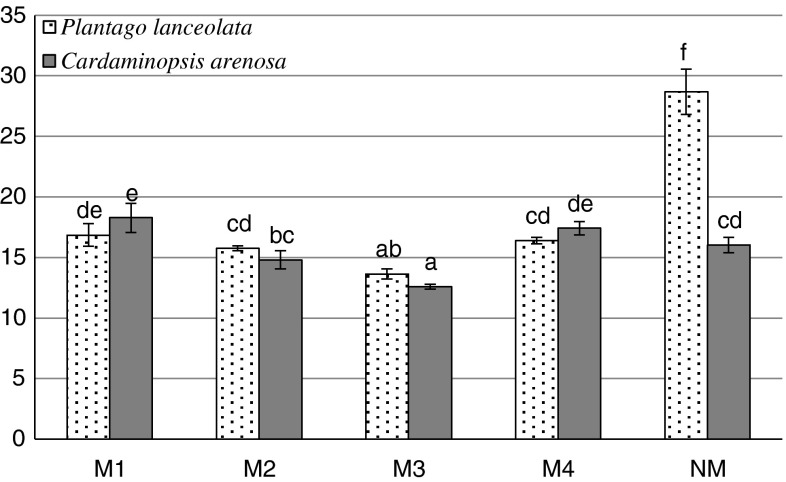
Proline content in *P. lanceolata* and *C. arenosa* leaves

An opposite tendency was recorded for non-protein thiols. Their levels were mostly lower in the leaves of plants that were exposed to metal contamination compared with the NM area (with the exception of *P. lanceolata* in M1 site) (Fig. 2), ranging from 137 to 311 and 248–578 nmol—SH g^−1^ fresh weight in *C. arenosa* and *P. lanceolata* respectively (Fig. 2). Non-protein thiol concentration in *P. lanceolata* leaves positively correlated with Cu and Pb contents, in opposition to a negative relationship between Cd or Fe contents and non-protein thiols levels in *C. arenosa* leaves (Tables 5, 6). We detected a decrease of proline concentration in the leaves of *P. lanceolata* in contaminated areas in comparison with the non-contaminated areas. However, we detected an increasing tendency in 50 % of cases for *C. arenosa* in contaminated areas (Fig. 3). The proline content negatively correlated with Fe, Mn Cu concentrations in the leaves of *C. arenosa* plants and with Cd, Zn, Pb and Mn in the leaves of *P. lanceolata* (Tables 5, 6).

**Fig. 4:**
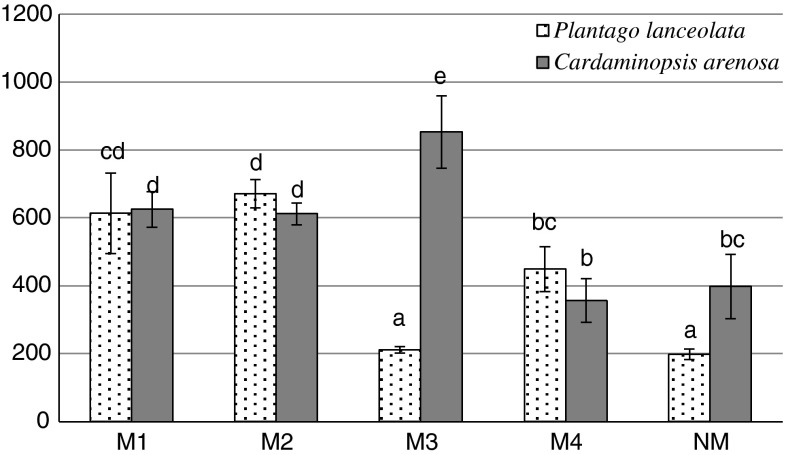
POD activity in *P. lanceolata* and *C. arenosa* leaves

The activities of POD were higher in most cases in plants exposed to heavy metals in soil than in those from the NM area. Only in the *C. arenosa* leaves from M4 site and *P. lanceolata* leaves from M3 site was the POD activity comparable with the activity in plant leaves from the NM area. The activity of POD ranged from 356 to 852 μmol tetra-guaiacol min^−1^ mg protein^−1^ in leaves of *C. arenosa* and 198–670 μmol tetra-guaiacol min^−1^ mg protein^−1^ in *P. lanceolata* (Fig. 4).

**Fig. 5:**
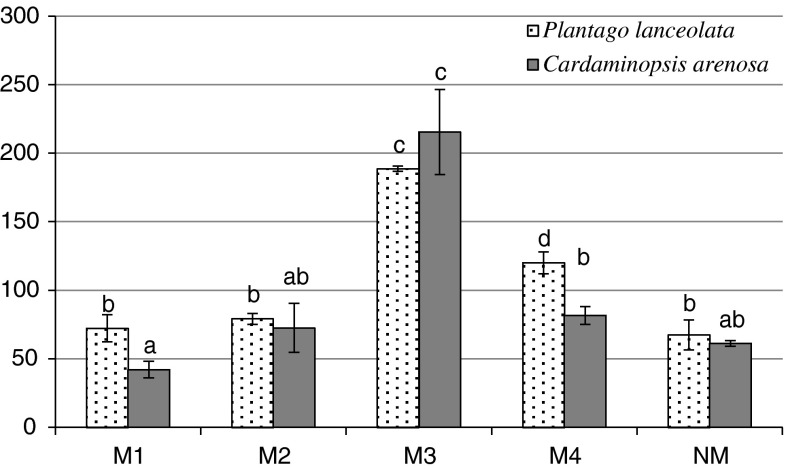
SOD activity in *P. lanceolata* and *C. arenosa* leaves

Superoxide dismutase activity was higher in the case of *C. arenosa* leaves collected at the M3 site (Fig. 5) than SOD activity in plant leaves at the NM site. We obtained similar results for *P. lanceolata,* with a higher SOD activity in the leaves of this plant collected at M3 and M4 sites than in plants from the NM area (Fig. 5).

Superoxide dismutase activity positively correlated with the Fe and Mn concentrations in the leaves of both species, and with Cu in *C. arenosa* leaves (Tables 5, 6). A negative correlation was found between the Cu concentration and SOD activity in *P. lanceolata* leaves (Table 6).

**Fig. 6:**
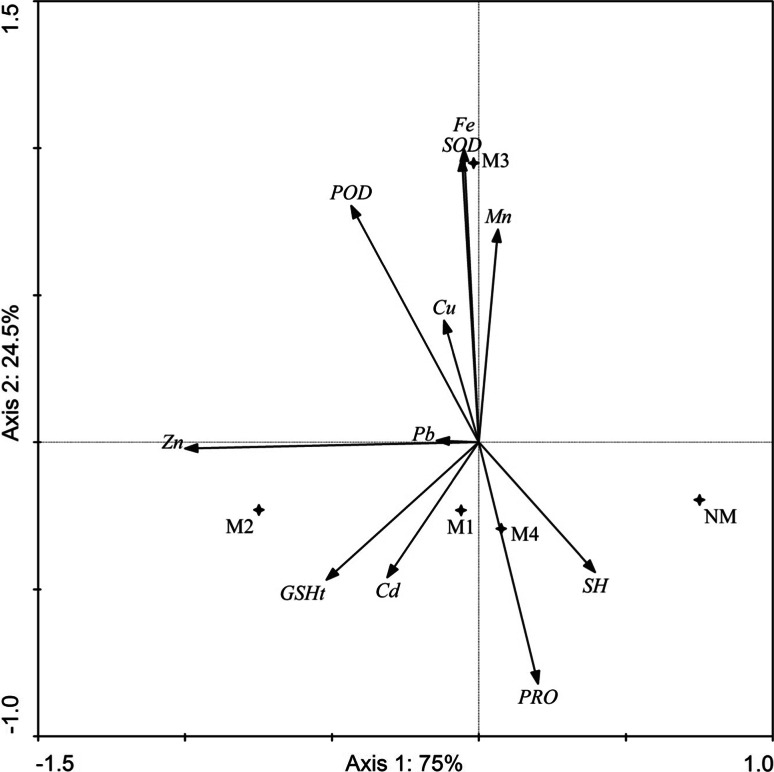
Principal component analysis performed on *C. arenosa* biochemical parameters and elements contents in the plants on investigated areas

**Fig. 7:**
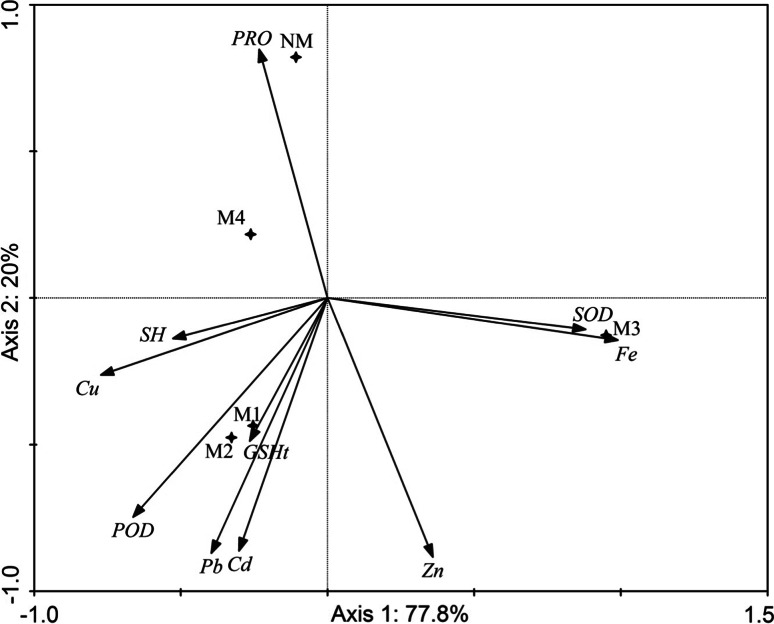
Principal component analysis performed on *P. lanceolata* biochemical parameters and elements contents in the plants on investigated areas

In *C. arenosa,* the first two axes of PCA explained 99.5 % of physiological characteristic variability (75 % by axis 1; 24.5 % by axis 2) (Fig. 6). Similarly, in *P. lanceolata,* the first two axes of PCA explained 97.8 % of physiological characteristic variability (77.8 % by axis 1, 20.0 % by axis 2) (Fig. 7).

In *C. arenosa*, the left side of the diagram in Fig. 6 presents samples collected in most of the contaminated areas. Also identified is a strong association between SOD and Fe, Cd and GSHt, Pb and Zn. In the case of *P. lanceolata* at contaminated sites, there was a strong relationship between POD and GSHt, Pb and Cd, SOD and Fe, and between non-protein -SH groups and Cu.

## Discussion

In this study we were interested in evaluating the physiological characteristics of heavy metal bioaccumulation capacity in two metallophytes, *C. arenosa* and *P. lanceolata,* growing in contaminated and non-contaminated areas.

Previously, Bidar et al. (2007) and Słomka et al. (2008) emphasized that many experiments are conducted on cultivated plants in laboratory conditions. Our results provide information about anti-oxidative defense in common plants, such as ruderals, widespread on metalliferous soil—*C. arenosa* and *P. lanceolata.* We also assessed heavy metal bioavailability in contaminated areas in comparison to a non-contaminated area.

The soil–plant transfer of chemical elements is part of nature’s element cycling. Total concentration of elements in soil cannot be considered as a good indicator of bioavailability. Bioavailable metals estimation allows the assessment of a plant’s potential to mobilize or bioaccumulate metals from soils (Braquinho et al. 2007). There is extensive literature concerning heavy metal contamination and exchangeable metal content or potential bioavailability in soil in the vicinity of smelting operations, dumps and mining areas (Liu et al. 2004; Chojnacka et al. 2005; Menzies et al. 2007). In our study, a higher concentration of (acid extracted) examined metals was found in calamine areas (M3 and M4) in comparison to the vicinity of the contamination emitters—M1 and M2. Cd and Zn were the most abundant among the examined metals. The bioavailable concentrations of Cd, Zn and Mn were higher in metalliferous soil near the smelting operation (M1and M2) than in soil near the mining area (M3 and M4). In a study by Majewska et al. (2011) on soil from a Zn-Pb waste heap in Bolesław, following 0.1 M NaNO_3_ extraction, the bioavailable concentration of Zn and Cd was less than 0.5 %. In our study, bioavailability results for the calamine sites (M3 and M4) were similar to Majewska et al. (2011) for Cd but lower for Zn (1.6–2.4 times). The bioavailability of these metals in soil near the smelting operations M1 and M2 was higher in our study and ranged from 1.1 to 12.4 % of metal amounts that were acid extracted. In addition, Moreno-Jiménez et al. (2009) emphasized that Cd and Zn usually are more mobile in soil than Cu. In their study, the percentages of extractable metals in relation to total soil metals showed that Cd and Mn were significantly more extractable than other metals in soils surrounding an abandoned mine in NW Madrid, Spain. In addition, Lei et al. (2010) showed that the proportions of water-soluble and exchangeable fractions extracted by selected analytical methods were the lowest among all gathered fractions in garden and paddy soils from a Pb/Zn mining area, similar to the results obtained by Banásowá et al. (2006) in former mining regions in central Slovakia.

Metal-tolerant plant species (metallophytes) maintain good performance as they are able to cope with higher internal metal levels due to adaptive genetic changes. However, successful colonization of highly metal enriched soils demands a *de novo* and in situ evolution of metal tolerance (Ernst 2006). In this study, examined specimens of *C. arenosa* and *P. lanceolata* came from metalliferous and non-metalliferous populations. Among metallophytes, pseudometallophytes, such as the species mentioned above, are attractive models for studying phenotypic differentiation driven by natural selection. In highly contaminated sites extreme environmental conditions may promote rapid differentiation between metalliferous and non-metalliferous populations (Meyer et al. 2010).

Herein, we compare the obtained results of metal bioaccumulation in the leaves of the examined species with the results of field studies on various plant species and their bioaccumulation abilities. The aim was to show the significance of information obtained directly from the environment not only in the bioindication of contamination but also in the potential use of individual plant species in phytomanagement of metal-contaminated soil. Bech et al. (2012) emphasizes that metal contaminated areas are potential sources of contamination due to wind and water erosion. Efforts to restore a vegetation cover can benefit stabilization and contamination control, and improve aesthetical aspects.

Gjorgieva et al. (2011) evaluated different plant organs from *Urtica doica, Taraxacum officinale*, *Matricaria recutita* and *Robinia pseudoacacia* as possible bioindicators of heavy metal contamination in the Republic of Macedonia. *T. officinale*, *U. dioica* and *R. pseudoacacia* were the highest accumulators. The maximum Pb content (in *R. pseudoacacia*) was 174.52 mg kg^−1^ in the Veles area where Pb and Zn metallurgical activity were present, whereas maximum Zn concentration for *U. dioica* leaves sampled near a lead smelting plant was 465 mg kg^−1^. These bioaccumulation levels are similar to the amounts of Zn and Pb recorded for the leaves of *P. lanceolata* sampled in a contaminated area in our study.

Moreno-Jiménez et al. (2009) investigated heavy metal transference to wild flora at an abandoned mine site in NW Madrid, Spain. The ranges of bioaccumulated heavy metals (mg kg^−1^) in annual and perennial herb shoots were 1.28–14.51 mg kg^−1^ for Cd, 59.18–581.1 mg kg^−1^ for Zn, 2.77–23.5 mg kg^−1^ for Cu, 46.5–230.2 mg kg^−1^ for Mn, 39.5–422.1 mg kg^−1^ for Fe. The bioaccumulation ranges were similar to heavy metal bioaccumulation results in our study, especially for *P. lanceolata* leaves. In this study, Cd and Zn were higher in *C. arenosa* in contaminated areas. However, the results of both our and Moreno-Jiménez et al. (2009) investigations correspond with the statement that in metalliferous soils there are gradients of plant-available metal levels which are reflected in the gradient of metal tolerant individuals (Ernst 2006).

Majewska et al. (2011) examined above-ground parts of *Biscutella laevigata* from the Bolesław heaps and determined 4,581 mg kg^−1^ Zn, 447 mg kg^−1^ Pb and 58 mg kg^−1^ Cd. Similar amounts of Cd but lower Pb and Zn concentrations were found in a higher bioaccumulating plant—*C.arenosa*—in our investigations. Szarek-Łukaszewska and Niklińska (2002) reported that in plants growing on calamine dumps near Olkusz, maximum metal concentrations in roots and shoots of *B. laevigata* were 14.3 mg kg^−1^ Cd, 111 mg kg^−1^ Pb and 410 mg kg^−1^ Zn. *P. lanceolata* contained up to 65.6 mg kg^−1^ Cd, 157 mg kg^−1^ Pb and 2,540 mg kg^−1^ Zn. Dimitrova and Yurukova (2005), after research on *P. lanceolata* in contaminated (vicinity of non-ferrous smelting works) and from sites away from major sources of contamination, emphasized that *P. lanceolata* leaves can be used as bioaccumulative indicators not only for Zn and Pb but also Cd. However, in our study the leaves of *C. arenosa* were better bioaccumulative indicators for these metals. In our investigations, *C. arenosa* better reflected soil heavy metal concentrations. Also Kucharski et al. (2005) described *C. arenosa* bioaccumulating properties. In a mesocosm experiment with highly metal contaminated soil from a non-ferrous mine and metal smelting site in southern Poland, and soil with calcium phosphate as a heavy metal-stabilizing amendment, *Deschampsia caespitosa* and *C. arenosa* were examined (Kucharski et al. 2005). *C. arenosa* was found undesirable for phytostabilization as it bioaccumulated high amounts of Zn and Cd in its shoots even though it provided better growth cover than *D. caespitosa* in soil without metal stabilizing amendment (Kucharski et al. 2005).

To evaluate environmental risk assessment, the obtained concentrations in the leaves of examined specimens were compared with metal concentrations mentioned as toxic for plant tissues. In our investigation, the measured concentrations of Cd and Zn in *C. arenosa* leaves generally were above toxic ranges in the metalliferous populations (Tables 2, 7). Pb contents in *C. arenosa* leaves, and Cd, Zn and Pb concentrations in *P. lanceolata* leaves, were in the ranges mentioned as toxic for plant tissues (Tables 2, 3, 7). Cd concentrations in the leaves of *C. arenosa* from M4 site were in the cut-off range of contents mentioned for hyperaccumulators (Tables 2, 7). The Cd and Zn bioaccumulation efficiency in *C. arenosa*, a plant with higher bioaccumulation, was also compared with the accumulation capacity of hyperaccumulators. In comparison to hyperaccumulators only plants from the metalliferous *C. arenosa* population at the M2 site bioaccumulated 50 % of the Zn amount bioaccumulated by hyperaccumulators (Alloway and Ayres 1999; Kabata-Pendias 2001; Serbula et al. 2012). However, Nouri et al. (2009) suggested that metal concentrations higher than toxic level in some species indicate that internal detoxification metal tolerance mechanisms might also exist, and therefore plant utility for phytoremediation is possible. Our results for the species studied also confirm this statement. Additionally, Yoon et al. (2006) and Massa et al. (2010) postulated that the identification of metal-tolerant plant species from natural vegetation in field sites and vegetational characterisation in contaminated sites, is relevant under an ecological point of view and even more when considering the plant species as accumulators in phytoremediation plans. As emphasized by Gonzáles et al. (2006) and Bech et al. (2012) metal concentrations in wild plants may vary. Tolerant species are able to grow in highly contaminated substrates, some of them behave as excluders and store the excessive levels of trace elements that may enter their roots, thereby protecting the more sensitive tissues from toxicity. Previously published data show that *P. lanceolata* is an excluder species (Szarek-Łukaszewska and Niklińska 2002). However, further investigations, also under controlled conditions, are needed to confirm if the studied species are indeed excluders (Gonzáles et al. 2006). In addition, phytostabilization requires metal tolerant species that are efficient in both rapid coverage of the contaminated soil and exclusion of metals from plant parts that are consumed by herbivores (Bech et al. 2012).

**Table 7:** Heavy metals concentration previously reported as toxic for plants, cut-off of heavy metals concentration used to define plant hyperaccumulators in comparison to heavy metals allowable concentration in soil (in mg∙kg^−1^)

	Toxic concentration in plants^a,c^	Hyperaccumulation limits^a^	Allowable Concentration in soil^b^
Cd	5–30	100	4
Zn	100–400	10,000	300
Pb	30–300	1,000	100
Cu	20–100	1,000	150
Mn	400–1,000	10,000	–
Fe	–	10,000	–

^a^Massa et al. (2010)

^b^The regulation of Environment Minister about the standards of soil and ground quality (2002)

^c^Kabata-Pendias (2001)

In our study, we also compared the anti-oxidative response in examined plant species that are widespread in contaminated areas. Information on the changes in the activity of oxidative enzymes obtained from field studies may be helpful for using them as biomarkers in the future and also may help better understand the defense mechanisms of plants experiencing chronic heavy metal stress and for research connected with revegetation of heavy metal contaminated sites. We proved that GSHt was increased in most investigated individuals of *C. arenosa* and *P. lanceolata* in metalliferous areas. GSH participates in the control of H_2_O_2_ levels in plant cells (Foyer and Noctor 2005), and plays a fundamental role in many cellular detoxification processes of xenobiotics and heavy metals. GSH does this by prior activation and conjugation with such compounds. Next to quenching of ROS during metal exposure, GSH acts as a precursor for the synthesis of phytochelatins (PCs) (Yadav 2010). Hawrylak and Szymańska (2004) described reduced glutathione as the most abundant non-protein thiol; however, in plant cells, other low-molecular weight compounds containing –SH groups can exist, such as PCs (phytochelatins), MTs (metallothioneins), thionins and defensins. Molecules containing sulfur, which exist in a wide variety in cells, may fulfill different functions and may be independently regulated (Mishra et al. 2009). In our study GSHt was positively related with Zn and Cd concentration in *C.arenosa* and with Pb concentration in *P. lanceolata* (Tables 5, 6; Figs. 6, 7). Conversely, the –SH group content was lower in most cases of investigated plants in metalliferous areas. In our previous field study on *Philadelphus coronarius* collected from contaminated areas, an increase of glutathione was found in leaves not infested with aphids in contaminated areas, and similar to this study the content of –SH groups was lower or was at a comparable level in comparison to non-contaminated areas (Kafel et al. 2010). Additionally, Boojar and Tavakkoli (2011) described that a pioneer plant species, *Zygophyllum fabago*, in comparison to *Peganum harmala*, grown in tailings of a Pb and Zn mine, showed an increase of PCs and GSH levels in aerial parts. Pongrac et al. (2009) found that Cd amendment decreased total chlorophyll concentration and glutathione reductase activity but increased non-protein thiol concentration in less tolerant *Thlaspi praeox* in comparison to *T. caerulescens*.

Our present study found a lower proline concentration in leaves of *P. lanceolata* in contaminated area. However, we also found a comparable proline level in most cases of metalliferous *C. arenosa* populations. Proline accumulation in plants is reported during conditions of drought, high salinity, high light and UV irradiation, elevated heavy metal levels, oxidative stress and in response generally to biotic stresses. Proline has been shown to function as a molecular chaperone able to protect protein integrity and enhance the activities of different enzymes (Szabados and Savouré 2009). In many studies, an increase in proline in plants has been recorded during response to heavy metal stress (Sharma and Dietz 2006; Sun et al. 2007). An increase in free proline in environmental contamination was also found in *Philadelphus coronarius* (Kafel et al. 2010). On the other hand, in metallophytes like *Silene vulgaris*, constitutive proline content was higher in metal-tolerant ecotypes, whereas metal-induced proline accumulation was higher in a non-tolerant ecotype (Schat et al. 1997). An investigation of proline in *Brassica juncea* showed an elevated level at lower concentrations of Cd and Pb but at higher concentrations it decreased (John et al. 2009).

The antioxidant enzymes, especially SOD, CAT and POD are present in various cellular compartments, functioning at different steps of ROS degradation and removal (Mishra et al. 2006). It is known that SOD is the main antioxidant enzyme functioning as a superoxide radical scavenger in living organisms, a first line defender against oxidative stress caused by ROS. POD, in addition to CAT and ascorbate peroxidase (APX), is an enzyme involved in the removal of H_2_O_2_ (Słomka et al. 2008; Boojar and Tavakkoli 2011). The balance maintenance between ROS and the antioxidant system is crucial for survival and adaptation of plants which grown in soils with relatively constant level of heavy metals (Słomka et al. 2008). Research on activity of the antioxidant enzymes is important to better understand antioxidant protection, especially in metallophytes.

In our study a higher POD activity in most cases for *C. arenosa* and *P. lanceolata* was recorded on metalliferous sites as compared to non-metalliferous ones. Additionally, the POD activity positively correlated with the concentration of Cu, Fe and Mn in *C. arenosa* and with Pb, Cd, Zn and Cu in *P. lanceolata* leaves (Tables 5, 6; Figs. 6, 7). The resistance of plants to heavy metal stress may be associated with the decreased susceptibility of enzymes to metal inhibition (Liu et al. 2004). This statement was confirmed for POD activity. Higher SOD activity was found only in leaves of the studied plants on calamine sites—M3 (for both) and M4 (for *P. lanceolata*). An increase in SOD activity in leaves of both studied species was observed under influence of Fe and Mn (Tables 5, 6; Figs. 6, 7). Elevated SOD and POD activities in the leaves and roots of barley along with bioaccumulation of Al, Cd and Cu were observed by Guo et al. (2007). An elevation of POD activity in *P. coronarius* leaves was found by Kafel et al. 2010. Boojar and Tavakkoli (2011) describe the elevation of SOD in aerial parts of *Z. fabago* in a metal-contaminated area (Zone1) as well as a comparable level of SOD activity of another species, *P. harmala*. Five species, including *Arrhenaterum eliatus, Bromus tectorum, Euphorbia cyparissias, Hypericum perforatum* and *Tanacetum vulgare* were examined by Dazy et al. (2009) in an ecotoxicological study of contaminated wasteland communities at a former coke factory in France. A clear dose–effect relationship was observed for SOD activity for all these species, POD presented bell-shaped dose–response curves. In a similar study on *Erigerion canadensis, Matricaria recutita* and *Oenothera biennis* in a contaminated area (also a former coke factory), SOD and POD activity exhibited higher levels in *E. canadensis* and *M. recutita* in contaminated areas compared to control (Dazy et al. 2008). In the leaves of *Viola tricolor* from one of the metalliferous areas of the Warpie heap in Chrzanów (southern Poland), SOD activity was higher than in other metalliferous and non-contaminated sites. Comparable or lower activity was characteristic for POD in the leaves of *V. tricolor* in metalliferous versus non-contaminated areas of Zakopane meadows (Słomka et al. 2008). The investigators emphasized that chronic metal-induced stress evokes a very similar mode of anti-oxidative response in all representatives of the populations and did not cause a measurable increase in oxidative stress. Differences in enzyme activity were the results of adjustment of the plants to different conditions (Słomka et al. 2008). In our study, a similar mode of antioxidant responses has been observed in plant leaves of metalliferous populations. This indicates the tolerance of these plants to heavy metals. However, POD and GSHt had a particularly strong role of in defense reactions, as their increase was the most common reaction to heavy metal contamination. Our main hypothesis was confirmed only for parameters such as POD and GSHt.

## Conclusion

*Cardaminopsis arenosa* (L.) Hayek and *P. lanceolata* L. have been used as model organisms and as examples of metallophytes because of their common presence throughout the studied areas. The levels of metals, especially Zn, Cd and Pb in the leaves of *C. arenosa* better reflected the metal concentrations in metalliferous and non-metalliferous soil than did the levels of metals in *P. lanceolata* leaves. The evaluated Cd concentration in the leaves of *C. arenosa* from the M4 site were in the range of heavy metal content suggested for hyperaccumulators. In metalliferous soils, there are gradients of bioavailable metal concentrations which are mirrored in varied metal tolerant individuals. We found several examples of high correlations between antioxidant parameters and concentrations of metals in soil and leaves of plants, and higher POD activity as well as GSHt content. POD and GSHt had a particularly strong role in heavy metal defense reactions. These relationships are characteristic of other field studies involving metallophytes. Generally, proline and thiol concentrations in plants in metalliferous areas were not elevated in relation to non-metalliferous areas. In conclusion, our results indicate that the ruderals *C. arenosa* (L.) Hayek and *P. lanceolata* L. may play an integral role in contaminated area stabilization and protection against heavy metal movement.

## Abbreviations

M: Metalliferous
NM: Non-metalliferous
POD: Guaiacol peroxidase
SOD: Superoxide dismutase
GSHt: Glutathione total
